# IKKβ activates p53 to promote cancer cell adaptation to glutamine deprivation

**DOI:** 10.1038/s41389-018-0104-0

**Published:** 2018-11-26

**Authors:** Mari B. Ishak Gabra, Ying Yang, Xazmin H. Lowman, Michael A. Reid, Thai Q. Tran, Mei Kong

**Affiliations:** 1 Department of Molecular Biology and Biochemistry, School of Biological Sciences, University of California, Irvine, Irvine, CA 92697 USA; 2 Department of Pharmacology and Cancer Biology, Duke University School of Medicine, Durham, NC 27708 USA

## Abstract

One of the hallmarks of cancer is the ability to reprogram cellular metabolism to increase the uptake of necessary nutrients such as glucose and glutamine. Driven by oncogenes, cancer cells have increased glutamine uptake to support their highly proliferative nature. However, as cancer cells continue to replicate and grow, they lose access to vascular tissues and deplete local supply of nutrients and oxygen. We previously showed that many tumor cells situate in a low glutamine microenvironment in vivo, yet the mechanisms of how they are able to adapt to this metabolic stress are still not fully understood. Here, we report that IκB-kinase β (IKKβ) is needed to promote survival and its activation is accompanied by phosphorylation of the metabolic sensor, p53, in response to glutamine deprivation. Knockdown of IKKβ decreases the level of wild-type and mutant p53 phosphorylation and its transcriptional activity, indicating a novel relationship between IKKβ and p53 in mediating cancer cell survival in response to glutamine withdrawal. Phosphopeptide mass spectrometry analysis further reveals that IKKβ phosphorylates p53 on Ser392 to facilitate its activation upon glutamine deprivation, independent of the NF-κB pathway. The results of this study offer an insight into the metabolic reprogramming in cancer cells that is dependent on a previously unidentified IKKβ–p53 signaling axis in response to glutamine depletion. More importantly, this study highlights a new therapeutic strategy for cancer treatment and advances our understanding of adaptive mechanisms that could lead to resistance to current glutamine targeting therapies.

## Introduction

The increased importance of glutamine uptake driven by oncogenes in cancer cells makes targeting glutamine metabolism an appealing approach for improved cancer therapy^1–3^. Glutamine, a non-essential amino acid, can be utilized by highly proliferative cancer cells to support cancer growth by replenishing the tricarboxylic acid (TCA) cycle intermediates, and providing a nitrogen source for the biosynthesis of other amino acids and nucleotides^4–6^. Moreover, glutamine can combat cellular oxidative stress as it supports the synthesis of the antioxidant, glutathione (GSH)^7^. However, as tumors continue to grow, increased glutamine demand and poor vascularization leads to its depletion in the microenvironment^8^. Multiple in vivo studies, including our recent publication, reveal that glutamine is among the amino acids depleted in the core of several xenograft tumors including melanoma, pancreatic adenocarcinoma, and colorectal cancer^9–11^. Therefore, cancer cells develop mechanisms to survive periods of nutrient starvation as new vascularization is developed. We recently reported that cancer cells are able to survive glutamine deprivation through the activation of cell cycle arrest genes mediated by p53, or metabolic reprogramming of glycolytic enzymes, but other mechanisms can also contribute to cell survival^12,13^. Thus, understanding the molecular mechanisms of how cancer cells attain this metabolic reprogramming and promote survival in a glutamine poor environment need to be fully understood in order to increase the efficacy of targeting glutamine metabolism therapeutically.

Recently, several studies have shown that the tumor suppressor p53 has a critical role in the aberrant metabolism in cancer and can orchestrate cellular adaptions to metabolic stress^14–17^. For instance, p53 was shown to upregulate oxidative phosphorylation and modulate antioxidants in lung cancer cells in response to glycolytic stress^18^. This role was further demonstrated by the activation of p53 upon glucose starvation and its regulation of TIGAR, a novel regulator of glycolytic genes, in response to this metabolic stress^19^. Similarly, serine or glutamine deprivation have been shown to activate p53 to promote survival through the induction of downstream genes such as the cyclin-dependent kinase inhibitor, p21^13,20^. Thus, it has become apparent that p53 acts as a master metabolic regulator, which can promote cancer cell survival in response to metabolic stress through multiple mechanisms.

The activation of the I-kappa-B-kinase (IKK) complex and the nuclear factor kappa B (NF-кB) subunits is implicated in the inflammatory response, cell survival, and cancer^21–23^. Despite the homology between the IKK complex kinases, IKKα and IKKβ, the IKKβ subunit is required for the rapid activation of NF-кB in response to stimuli, and is shown to phosphorylate other substrates directly, such as BAD, p85α, and β-catenin, independent of the IKK complex^24–26^. Recent studies reveal a role for IKKβ in sensing metabolic stress. For instance, IKKβ is activated upon leucine starvation and promotes feedback inhibition of the PI3K/AKT signaling pathway^27^. Moreover, the IKK complex regulates oxidative phosphorylation in normal and cancer cells by upregulating mitochondrial synthesis of cytochrome *c* oxidase 2 when glucose levels are low^28^. IKKβ is also activated upon glutamine deprivation, and inhibits PFKFB3, a major driver of glycolysis, to regulate cellular metabolic adaptation^29^.

Even though several studies demonstrate the activation of IKKβ and p53 under low glutamine conditions to promote cellular adaptation, the mechanism of how these major metabolic sensors interact to promote cell survival upon metabolic stress remains unknown. Here, we show that IKKβ modulates the activity of p53 in response to glutamine depletion to promote cancer cell adaptation. We further demonstrate that IKKβ phosphorylates p53 on Ser392 to enhance its transcriptional activity, independent of the NF-κB pathway. Our data provide a mechanistic insight into the role of an IKKβ–p53 signaling axis that mediates cancer survival in the nutrient-deprived tumor microenvironment.

## Results

### Glutamine deprivation induced p53 activation is IKKβ dependent

**Fig. 1:**
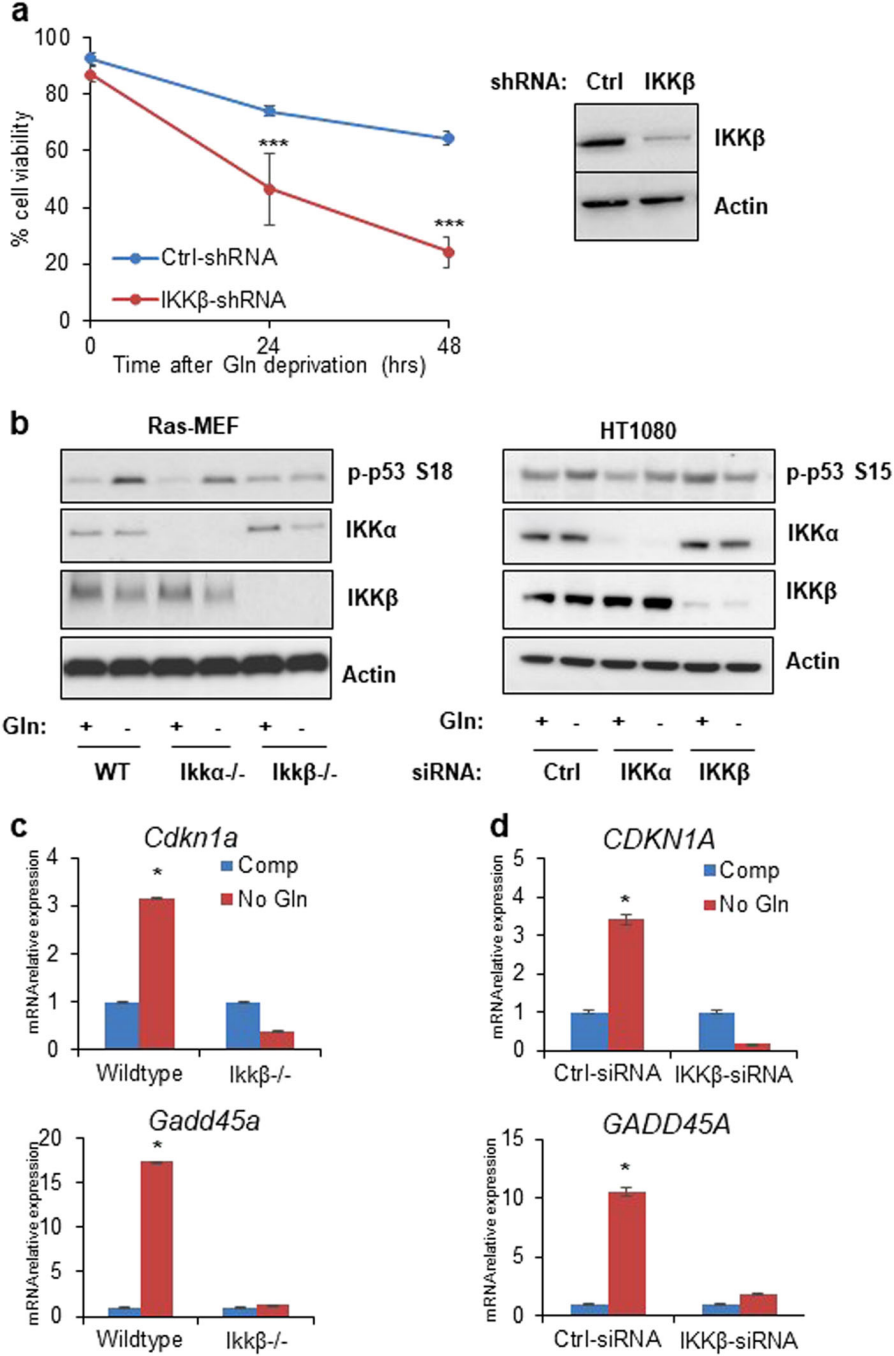
Glutamine deprivation induced p53 activation is IKKβ dependent. **a** HT1080 transduced with control (Ctrl) or IKKβ shRNA were cultured in glutamine-free medium for 24 and 48 h. Cell viability was assessed by PI exclusion. Cell lysate was collected for western blotting with the indicated antibodies. **b** Western blot analysis of wild-type (WT), *Ikkα*^−/−^ or *Ikkβ*^−/−^ MEFs and HT1080 cells transiently transfected with siRNA against scrambled control, IKKα, or IKKβ cultured in complete and glutamine (Gln)-free medium 24h and cell lysate was used for immunoblotting using the antibodies indicated. **c** Wild-type and *Ikkβ*^−/−^ MEFs were cultured in complete (Comp) or glutamine-free (No Gln) media overnight and mRNA was extracted for qPCR analysis of p53-target genes. **d** HT1080 cells transiently transfected with siRNA against scrambled control or IKKβ were cultured in complete or glutamine-free media overnight. mRNA was extracted, and expression of p53-target genes was determined using qPCR. **a**, **c**, and **d** represent means ± s.d. of triplicates from three independent experiments (*p < 0.05, **p < 0.01, ***p < 0.001 using Student’s *t*-test)

Previously, we reported that IKKβ is phosphorylated at Ser177 in response to low glutamine to downregulate glycolysis^29^. To further confirm the role of IKKβ in mediating cell survival, we stably knocked down IKKβ in HT1080 cells using shRNA and cultured IKKβ-deficient and control cells in glutamine-free medium. Consistently, we found that IKKβ-deficient cells were more sensitive to glutamine deprivation (Fig. 1a). Since p53 is known to be phosphorylated in response to glutamine deprivation and its phosphorylation is reversed by the use of antioxidants^12^, we asked whether IKKβ and p53 are acting synergistically to mediate cell survival. To test this, we cultured Ras-transformed 3T3 mouse embryonic fibroblast (MEF) cells with wild-type, *Ikkα*^−/−^ or *Ikkβ*^−/−^ alleles in glutamine-free medium for 24 h. p53 phosphorylation on Ser18 was observed in control and IKKα knockout cells in response to glutamine starvation. In contrast, the increase in p53 phosphorylation was blocked in IKKβ knockout cells (Fig. 1b). Consistently, HT1080 cells transiently transfected with siRNA against scrambled control, IKKα, or IKKβ and cultured in glutamine-free medium for 24 h show that only the loss of IKKβ decreased the levels of p53 phosphorylation at Ser15 (Fig. 1b). This attenuation in p53 phosphorylation in IKKβ-deficient cells prompted us to investigate whether the loss of IKKβ affected the transcription activity of p53. Thus, we evaluated the expression levels of *CDKN1A* and *GADD45A* in MEF and HT1080 cells cultured in glutamine-free medium. Interestingly, we found significant induction of these p53-target genes in control cells that is lost in IKKβ-deficient cells (Fig. 1c, d), suggesting the requirement for IKKβ in p53 activation under low glutamine conditions.

### Mutant p53 activation is also dependent on IKKβ under low glutamine

**Fig. 2:**
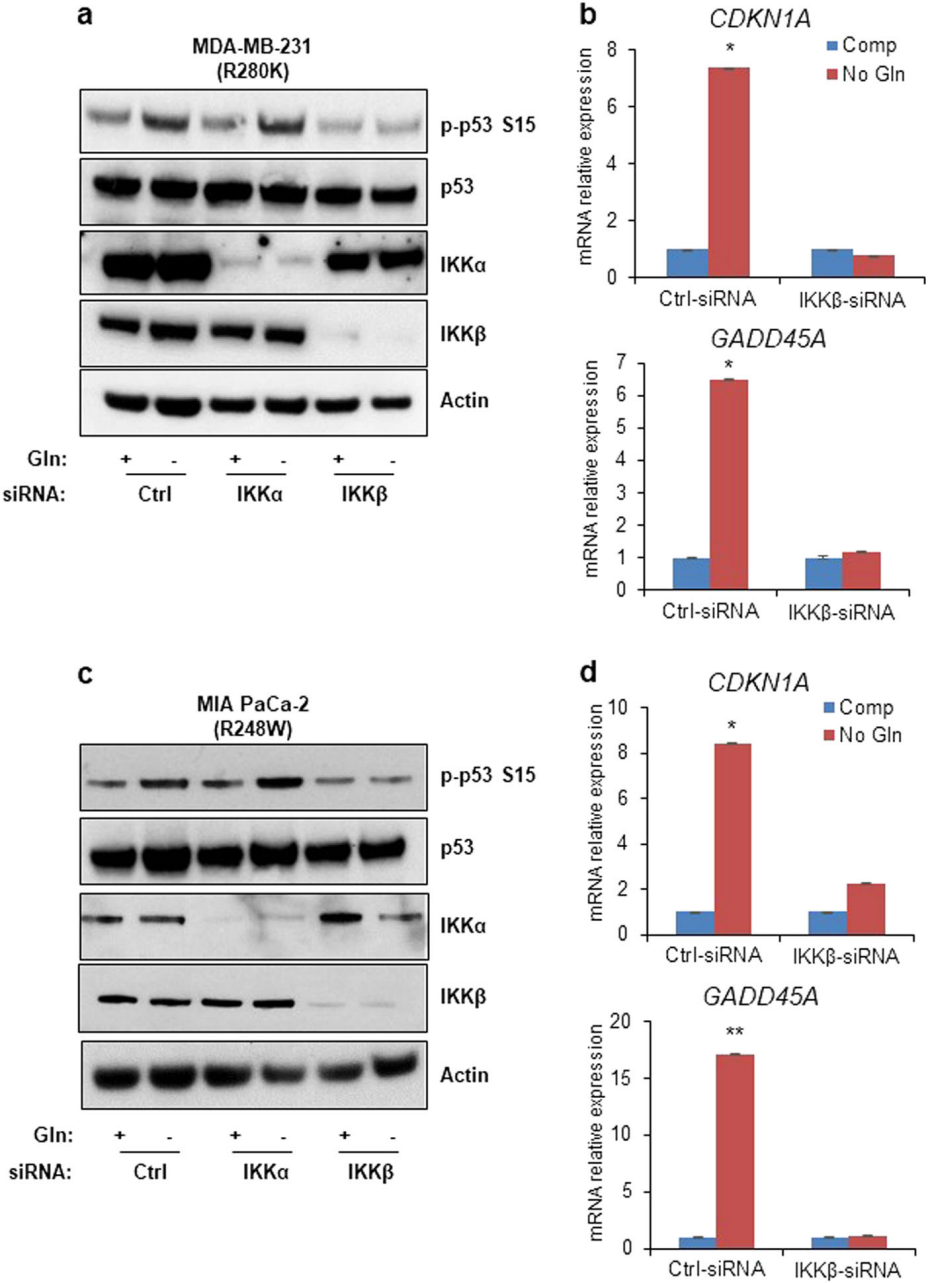
Mutant p53 activation is also dependent on IKKβ under low glutamine. **a**, **c** Western blot analysis of MDA-MB-231 and MIA PaCa-2 cells transiently transfected with siRNA against scrambled control (Ctrl), IKKα, or IKKβ cultured in complete and glutamine (Gln)-free media overnight. Cell lysate was used for immunoblotting using the antibodies indicated. **b**, **d** MDA-MB-231 and MIA PaCa-2 cells transiently transfected with siRNA against scrambled control or IKKβ were cultured in complete (Comp) or glutamine-free (No Gln) media overnight. mRNA was extracted, and expression of p53-target genes was determined using qPCR. **b** and **d** represent means ± s.d. of triplicates from three independent experiments (*p < 0.05, **p < 0.01, ***p < 0.001 using Student’s *t*-test)

The p53 protein is mutated in over 50% of all human cancer, with a high prevalence in mutations of the DNA-binding domain^30,31^. More importantly, mutant p53 (mutp53) has been shown to gain oncogenic functions through its transcriptional activity, which promotes cancer tumorgenicity^32^. We have previously shown that mutp53 is phosphorylated upon glutamine deprivation and subsequently leads to the induction of *CDKN1A*, which was necessary for the survival of cancer cells^13^. Therefore, we tested two cell lines with mutation in p53, the triple-negative breast cancer cell line, MDA-MB-231, and the pancreatic cancer cell line, MIA PaCa-2, with Arg280 and Arg248 mutation respectively to see whether loss of IKKβ affects the activation of mutp53. Similar to HT1080 cells, we transfected both MDA-MB-231 and MIA PaCa-2 cells with siRNA against scrambled control, IKKα, and IKKβ, and incubated cells in control and glutamine-free medium overnight. Strikingly, mutp53 activation by phosphorylation at Ser15 was lost in IKKβ knockdown cells but not in control or IKKα knockdown cells (Fig. 2a, c). This decrease in mutp53 phosphorylation was also accompanied by a loss in the induction of p53-target genes *CDKN1A* and *GADD45A* (Fig. 2b, d). These data further suggest an important correlation between IKKβ and p53 activation, indiscriminate of wild-type or mutant p53, upon glutamine deprivation.

### p53 activation upon glutamine deprivation is NF-кB independent

**Fig. 3:**
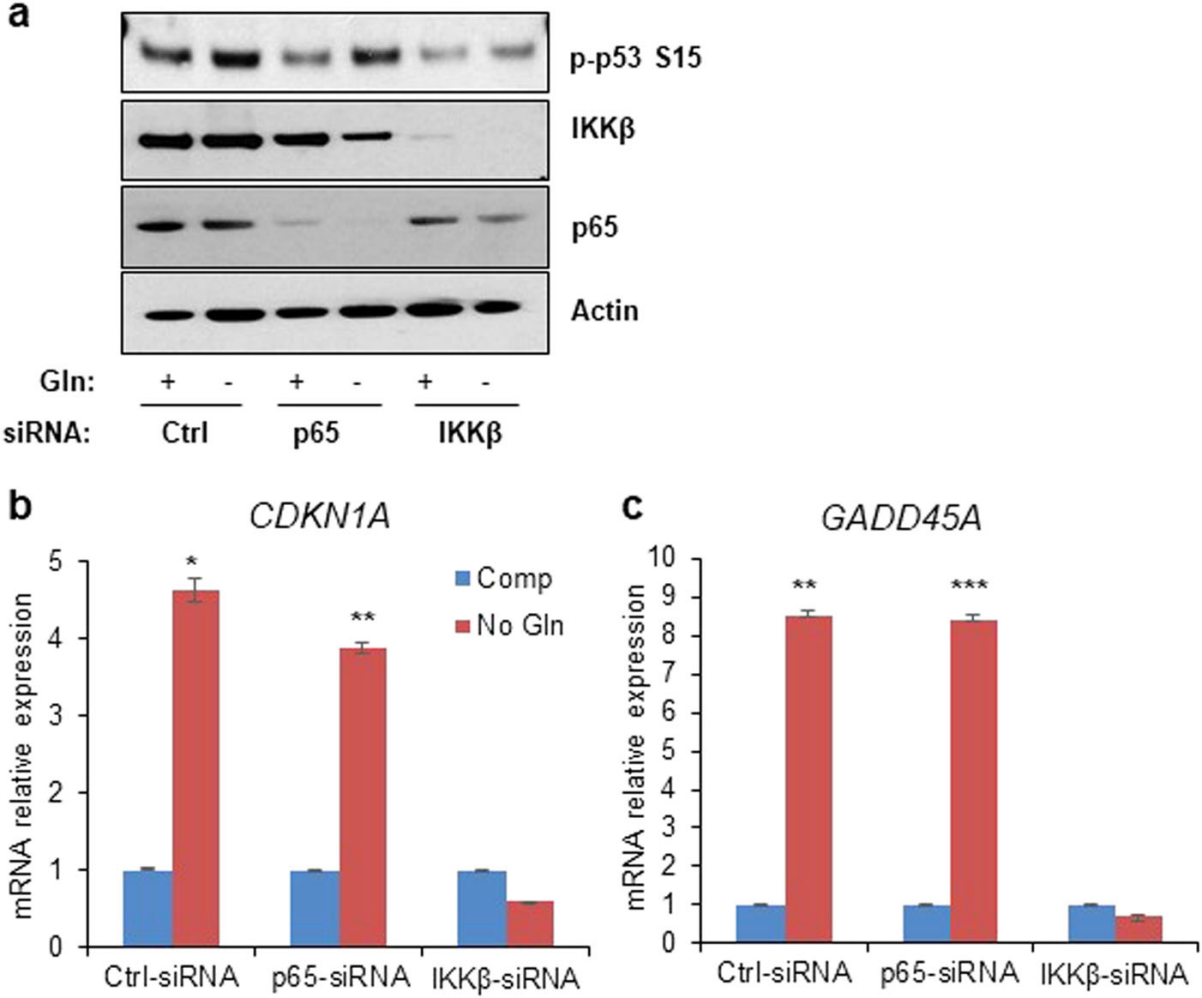
p53 activation upon glutamine deprivation is NF-кB independent. **a** HT1080 cells transiently transfected with siRNA against scrambled control (Ctrl), p65, or IKKβ cultured in complete or glutamine (Gln)-free media overnight. Cell lysate was used for immunoblotting using the antibodies indicated. **b**, **c** HT1080 cells transiently transfected with siRNA against scrambled control, p65, or IKKβ were cultured in complete (Comp) or glutamine-free (No Gln) media overnight. mRNA was extracted, and expression of p53-target genes was determined using qPCR. **b** and **c** represent means ± s.d. of triplicates from three independent experiments (*p < 0.05, **p < 0.01, ***p < 0.001 using Student’s *t*-test)

Next, we sought to determine whether IKKβ activated p53 through the NF-кB pathway. IKKβ is known to be a master regulator of several NF-кB subunits including RelA (p65), a dominant NF-кB transactivating subunit^33^. Therefore, we tested whether p65 knockdown affected p53 phosphorylation and induction of p53-target genes upon glutamine deprivation. Consistent with our previous results, the loss of IKKβ significantly attenuated the phosphorylation of p53. Interestingly, HT1080 transiently transfected with siRNA against p65 showed an increase in the phosphorylation of p53 upon glutamine withdrawal to a similar level as the control (Fig. 3a). Moreover, the expression of p53-target genes, *CDKN1A* and *GADD45A*, was significantly induced in p65 knockdown cells cultured in glutamine-free medium overnight, similar to control cells (Fig. 3b, c). Meanwhile, IKKβ knockdown cells lost this induction of p53 downstream genes. These data suggest that IKKβ is regulating the phosphorylation of p53 independent of NF-кB transactivation.

### IKKβ phosphorylates p53 on Ser392 in response to low glutamine

**Fig. 4:**
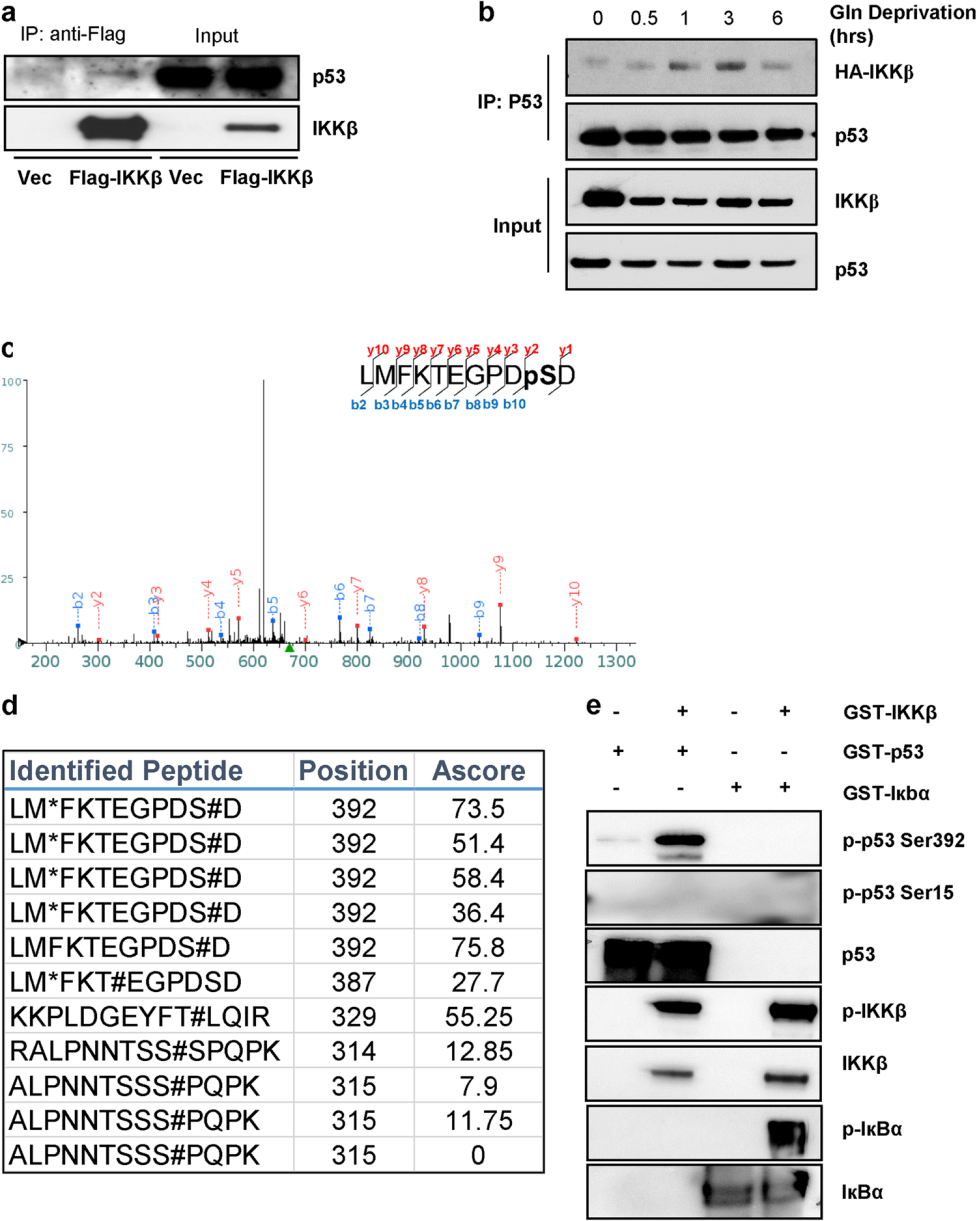
IKKβ phosphorylates p53 on Ser392 in response to low glutamine. **a** 293T cells transiently expressing vector (Vec) or Flag-IKKβ were cultured in glutamine-free medium overnight and immunoprecipitated with anti-Flag conjugated beads followed by immunoblotting with the indicated antibodies. **b** 293T cells transiently expressing HA-IKKβ and wild-type p53 were cultured in glutamine-free medium for 0, 0.5, 1, 3, and 6 h. Cell lysate was immunoprecipitated with anti-p53 antibody followed by immunoblotting with the indicated antibodies. **c** Kinase assay using recombinant GST-IKKβ and recombinant GST-p53 was performed followed by SDS-PAGE and phosphopeptide mapping. Mass spectra of identified phosphopeptide sequence on Ser392 of p53 is presented. **d** Identified phosphopeptides (as described in **c**) with positions on p53 and confidence score (A score) are presented. **e** Kinase assay using recombinant GST-IKKβ and recombinant GST-p53 or GST-IкBα (positive control) was performed followed by immunoblotting with the indicated antibodies

Since IKKβ has been previously shown to directly phosphorylate its substrates, we tested whether IKKβ could phosphorylate p53 upon glutamine deprivation. Thus, we overexpressed Flag-tagged IKKβ in 293T cells and starved cells of glutamine overnight followed by immunoprecipitation using anti-Flag conjugated agarose beads to purify IKKβ-interacting proteins. We found that p53 interacts directly with IKKβ, albeit rather weakly, which could be due to the transient nature of the kinase–substrate interaction (Fig. 4a). To further confirm this binding, we transiently co-expressed HA-tagged IKKβ and p53 in 293T cells and cultured these cells in glutamine-free medium. Cells were collected at 0, 0.5, 1, 3, and 6 h and lysates were immunoprecipitated using anti-p53 antibody. Interestingly, IKKβ interaction with p53 appeared as early as 1 h post glutamine withdrawal followed by its dissociation at 6 h (Fig. 4b). Next, to identify which residue on p53 is phosphorylated by IKKβ, we performed an in vitro kinase assay using recombinant GST-IKKβ, which is constitutively active, and recombinant GST-p53 followed by phosphopeptide mapping using mass spectrometry. We found that p53 was phosphorylated on Ser392 in the c-terminal domain (Fig. 4c, d). To confirm the phosphorylation of p53 on Ser392 by IKKβ, we performed an in vitro kinase assay followed by immunoblotting and found that IKKβ phosphorylated GST-IкBα, a known IKKβ substrate, and GST-p53 on Ser392, but not on Ser15 (Fig. 4e). Taken together, these results indicate that IKKβ phosphorylates p53 on Ser392 as an early response to glutamine deprivation and possibly later facilitates its phosphorylation at Ser15 and transcriptional activity.

### Low glutamine induces phosphorylation of p53 on Ser392

**Fig. 5:**
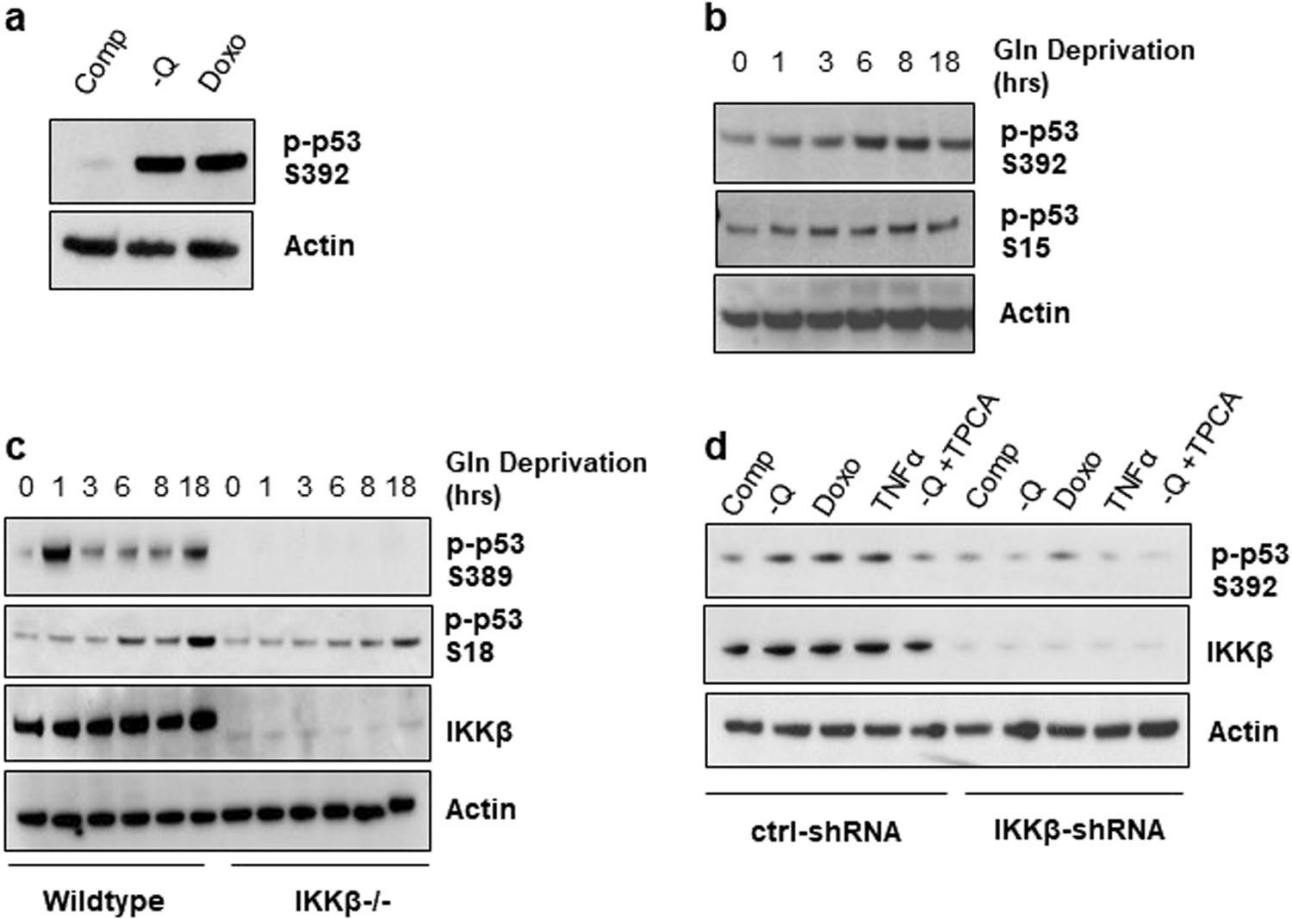
Low glutamine induces phosphorylation of p53 on Ser392. **a** HT1080 cells cultured in complete medium (Comp), glutamine-free (-Q) medium or complete medium with 2 μM Doxorubicin (Doxo) overnight. Cell lysate was collected for immunoblotting using the indicated antibodies. **b** Time course of p53 phosphorylation on Ser392 using HT1080 cells cultured in glutamine-free medium for 0, 1, 3, 6, 8, and 18 h. Cell lysate was collected for immunoblotting using the indicated antibodies. **c** Time course of p53 activation on Ser389 using wild-type and *Ikkβ*^−/−^ MEFs cultured in glutamine (Gln)-free medium for 0, 1, 3, 6, 8, and 18 h. Cell lysate was collected for immunoblotting using indicated antibodies. **d** HT1080 cells transduced with shRNA against control (Ctrl) or IKKβ were cultured in complete medium, glutamine-free medium, complete medium with 2 μM Doxo, or glutamine-free medium with 200 nM TPCA-1 (-Q + TPCA) for 6 h, or complete medium with 15 ng/ml TNFα for 15 min. Cell lysate was collected for immunoblotting with indicated antibodies

Phosphorylation of p53 on Ser392 is shown to increase in response to various stimuli, such as treatment with genotoxic inducing agents, UV irradiation, and the MDM2 inhibitor Nutlin-3a^34^. Therefore, to illustrate that phosphorylation of p53 on Ser392 is induced in response to glutamine deprivation, we cultured HT1080 cells in complete medium, glutamine-free medium, and complete medium with doxorubicin (Doxo), a DNA damaging reagent used as a positive control, and compared the phosphorylation of p53 on Ser392 by western blotting. Phosphorylation of p53 on Ser392 was induced in response to glutamine deprivation at a similar level to the genotoxic stress caused by Doxo treatment (Fig. 5a). Next, we wanted to evaluate the persistence of this phosphorylation with prolonged glutamine starvation by culturing HT1080 cells without glutamine and collecting cell lysate over the course of 18 h. Interestingly, phosphorylation on Ser392 seemed to increase with prolonged glutamine withdrawal, corresponding also with an increase in Ser15 phosphorylation, indicating its importance in mediating cellular response upon this metabolic stress (Fig. 5b). To determine the role of IKKβ in glutamine deprivation-induced p53 phosphorylation on Ser392, we compared Ras-transformed wild-type to *Ikkβ*^−/−^ MEF cells. Similarly, the phosphorylation of p53 on Ser389 (the analog of Ser392 in human p53) in wild-type cells seemed to increase with glutamine deprivation over time and was completely abolished by the loss of IKKβ in *Ikkβ*^−/−^ MEF cells (Fig. 5c). Concurrently, phosphorylation of p53 on Ser18 also increased over time upon glutamine deprivation. However, the level of p53 Ser18 phosphorylation in *Ikkβ*^−/−^ MEF cells was delayed, with a decreased level at 18 h compared to wild-type cells. Furthermore, the phosphorylation of p53 on Ser392 was dependent on IKKβ activity since it was induced in HT1080 cells treated with TNFα, which is known to activate IKKβ^35^, and was attenuated when cells were cultured in low glutamine with the addition of TPCA-1, an IKKβ inhibitor (Fig. 5d)^36^. Consistently, the phosphorylation on Ser392 was completely lost in HT1080 IKKβ knockdown cells treated with the same conditions (Fig. 5d). These results demonstrate that low glutamine conditions lead to the phosphorylation of p53 on Ser392 in an IKKβ-dependent manner.

### Phosphorylation of p53 on Ser392 is required for p53 activation upon glutamine deprivation

**Fig. 6:**
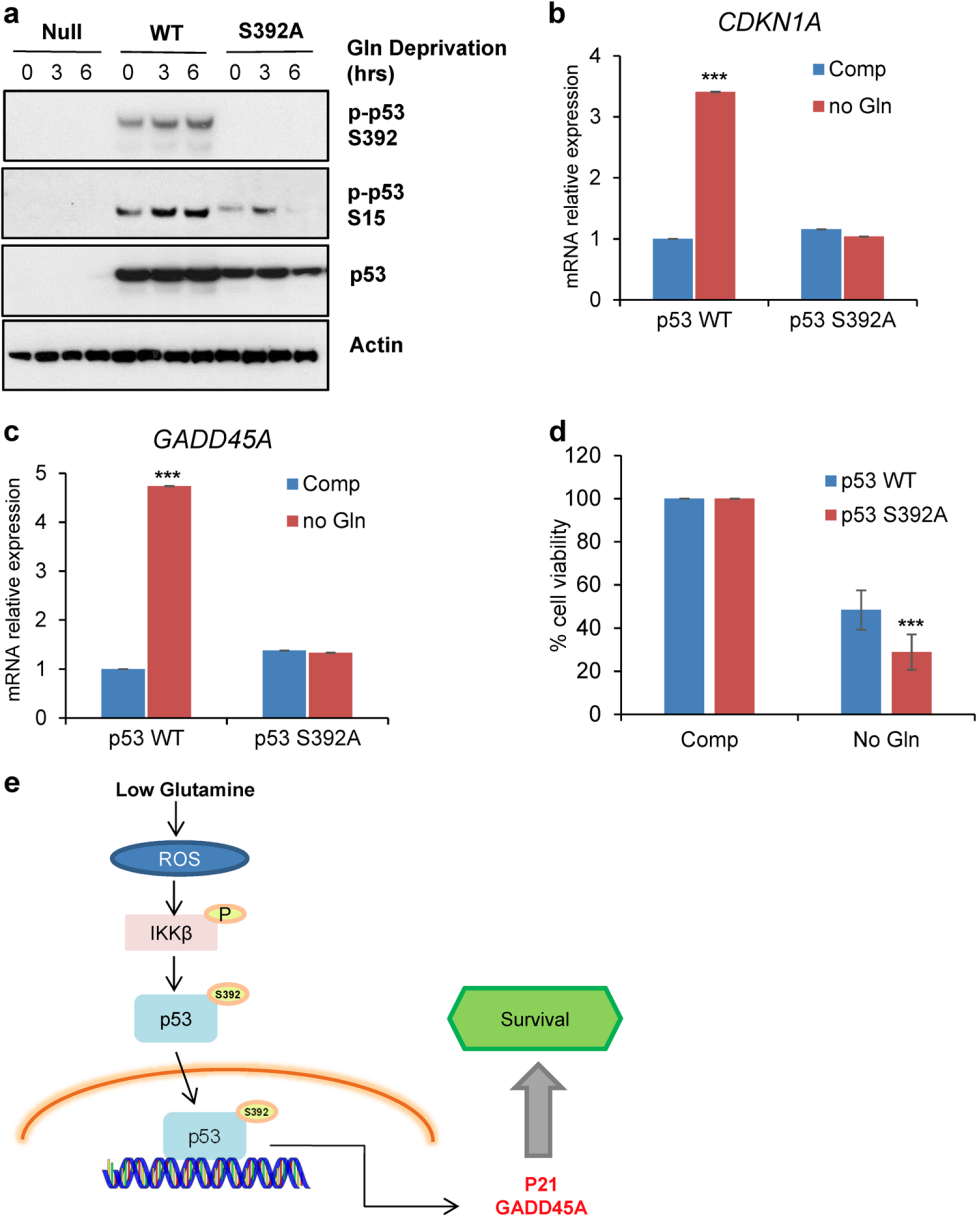
Phosphorylation of p53 on Ser392 is required for p53 activation upon glutamine deprivation. **a** Western blot analysis of p53-null PC3 cells and PC3 cells transiently expressing wild-type p53 (WT) or Flag-p53 S392A cultured in glutamine-free medium for 0, 3, and 6 h. Cell lysate was collected for immunoblotting with the indicated antibodies. **b**, **c** PC3 transiently expressing wild-type p53 (WT) or Flag-p53 S392A were cultured in glutamine-free medium for 6 h. mRNA was extracted, and expression of p53-target genes was determined using qPCR. **d** PC3 transiently expressing p53 WT or Flag-p53 S392A were cultured in glutamine-free medium for 48 h. Cell viability was assessed using trypan blue exclusion assay at time 0 or 48 h of glutamine deprivation. **e** Schematic showing molecular mechanism by which cells survive glutamine deprivation via the suggested IKKβ–p53 signaling axis. **b**, **c**, and **d** represent means ± s.d. of triplicates from three independent experiments (*p < 0.05, **p < 0.01, ***p < 0.001 using Student’s *t*-test)

To further investigate how Ser392 phosphorylation affects p53 activity upon glutamine deprivation, we ectopically expressed both wild-type and S392A-mutant p53 in the p53-null prostate cancer cells, PC3. We then cultured wild-type and S392A expressing PC3 cells along with null cells in glutamine-free medium and collected cell lysate at 0, 3, and 6 h. Interestingly, we found that the expression of the p53 point mutant, S392A, hampered the phosphorylation of p53 on Ser392 and Ser15 in response to glutamine withdrawal (Fig. 6a). We next assessed whether the mutation on Ser392 would affect the transcriptional activity of p53. Thus, we expressed wild-type and S392A p53 in PC3 and cultured these cells under no glutamine for 6 h. Consistently, the expression levels of *CDKN1A* and *GADD45A* increased upon glutamine deprivation in wild-type p53 expressing cells (Fig. 6b, c). However, this induction was attenuated by the mutation on Ser392, further suggesting that the phosphorylation of p53 on this residue has a role in the transcriptional activity of p53 in response to low glutamine. Next, we wondered if mutation on Ser392 would affect the role p53 has in survival. We expressed wild-type and S392A p53 in PC3 and assessed cell viability after 48 h in glutamine-free medium. Indeed, S392A p53 expressing cells had significantly less cell viability compared to wild-type (Fig. 6d). These data further support a model, in which activation of IKKβ in response to glutamine deprivation leads to the phosphorylation of p53 on Ser392, which is required for p53 activity in the transcription of downstream target genes and in promoting cancer cell survival (Fig. 6e).

## Discussion

IKKβ and the NF-кB pathway have been implicated in cancer cell response to metabolic stress and their activation is necessary for cancer cell survival^27^. Similarly, p53 has been shown to regulate glycolysis, cell cycle arrest, and expression of metabolic enzymes to promote survival in many cancer models^17,37,38^. As these pathways function to promote survival in cancer, it is critical to delineate whether IKKβ and p53 work synergistically or independently to combat metabolic stress or induce therapeutic resistance to drugs targeting glutamine metabolism. Here, we demonstrate a role for IKKβ in phosphorylating p53 upon glutamine depletion to promote cancer cell survival. Loss of IKKβ in cancer cells harboring both wild-type and mutant variants of p53 significantly reduced the phosphorylation of p53 and its transcriptional activity under low glutamine. Specifically, these results indicate that IKKβ can phosphorylate p53 on Ser392 in response to glutamine depletion and induce its transcriptional activity. Additionally, the mutation of Ser392 residue attenuated the activation of p53 and sensitized cancer cells to glutamine deprivation. This study highlights a new IKKβ–p53 signaling axis that is critical to cancer cell adaptation to metabolic stress.

Previously, there have been several studies indicating crosstalk between the IKKβ/NF-кB pathway and p53 stabilization and activation. For instance, under genotoxic conditions, IKKβ acts as a regulator of p53 via the association with β-TrCP1 in MEF cells to destabilize its protein levels, independent of Mdm2, and the loss of IKKβ via chemical inhibition in lung adenocarcinoma cells reduced proliferation and invasion in these cells, and was accompanied by the stabilization of p53^39,40^. Moreover, several studies link the activity of the DNA damage kinase, ATM, to the activation of the IKK complex and NF-кB under genotoxic stimuli^41,42^. These studies indicate the dynamic nature of p53 regulation by the IKK complex that is highly dependent on the type of stress. The realization that cancer cells experience metabolic stress in vivo prompted further investigation into the connection between the IKK pathway and p53. Interestingly, the activation of the NF-кB pathway is necessary to regulate p53 activity and protects cells against glucose starvation, while the loss of p53 leads to the upregulation of GLUT3 expression and glycolysis by the hyperactivation of NF-кB^21,28^. These studies further indicate the existence of a feedback loop connecting the activity of the IKKβ/ NF-кB pathway and p53 in modulating cancer metabolism. Therefore, the connection between IKKβ and p53, which are both activated in cancer, in response to glutamine starvation and other metabolic stress needs to be fully understood.

Post-translational modification of p53 is rapid and results in its stabilization in response to various stress stimuli^43^. The phosphorylation of p53 on the highly conserved residue, Ser392 (Ser389 in mice), was shown to have a role in site-specific DNA binding, formation of the p53 tetramer, and inducing cell arrest^44–46^. Additionally, a mutation of Ser392 to alanine was sufficient in disrupting p53 localization to the nucleus and its degradation in response to DNA damage^47,48^. Later studies indicated p53 has a basal level of phosphorylation on Ser392 in unstressed cells that increases when exposed to various stress conditions, such as irradiation, UV exposure, and etoposide treatment. To date, several kinases of Ser392 under genotoxic stress were identified such as CK2 and p38 MAPK, but other kinases that are resistant to CK2 inhibition remain to be identified^49–51^. More importantly, the phosphorylation of p53 on Ser392 in response to metabolic stress and the kinases that catalyze this phosphorylation remains to be elucidated. Our study provides an unprecedented signaling pathway connecting IKKβ to p53 phosphorylation on Ser392 in response to glutamine deprivation as a mechanism of survival in cancer cells.

The connection between the phosphorylation of p53 on Ser392 and Ser15 and their effect on p53 activity remain unclear in literature. p53 activation by Ser15 phosphorylation was observed in several cancer cell lines when cultured under metabolic stress. For instance, serine and glutamine withdrawal induced phosphorylation of p53 and its activation^12,20^. Similarly, glucose starvation and nutrient stress led to the activation of p53 by phosphorylation on Ser15^28^. Our findings suggest that the phosphorylation of p53 on Ser392 is required for its activity and role in cell survival upon glutamine withdrawal. Thus, it would be interesting to unveil whether Ser392 is also critical for p53 phosphorylation on Ser15 and its activity under different metabolic stress conditions. It is also possible that IKKβ could be activating p53 at Ser392 as an early response to glutamine deprivation followed by a later induction of Ser15 phosphorylation and p53 activity. However, more studies would be needed to further understand the regulation of p53 and its phosphorylation on both Ser15 and Ser392 in response to metabolic stress.

Our findings indicate that an IKKβ-p53 signaling axis could promote tumor growth despite glutamine deprivation. Furthermore, IKKβ knockdown affected the phosphorylation and activity of both wild-type and mutant p53 when cells were cultured in glutamine-free medium. As more in vivo studies reveal glutamine depleted niches due to rapid glutamine consumption by cancer or poor vascularization networks, it is important to understand how cells adapt to this metabolic stress in order to develop better therapeutic strategies. Therefore, future experiments will further investigate the attenuation of p53 phosphorylation by targeting IKKβ in vivo through combination treatment using IKKβ inhibitors and glutaminase inhibitors in cancer cells harboring wild-type or mutant p53. This study highlights the potential efficacy of a combination therapeutic strategy in targeting cancer growth, and identifies a potential resistance mechanism to ongoing clinical trials that use glutaminase inhibitors.

## Materials and methods

### Reagents and plasmids

Antibodies to IKKβ (8943), IKKα (2682), p-p53 S15 (9284), p-p53 S392 (9281), p-p65 (3033), p65 (3987), p-Iкbα (2859), and Iкbα (4814) were from Cell Signaling (Danvers, MA, USA), beta actin (A1978) was from Sigma (St. Louis, MO, USA), and p53 DO-1 (SC126) was from Santa Cruz Biotechnology (Dallas, TX, USA). Recombinant active GST- IKKβ (31176) was purchased from Active Motif (Carlsbad, CA, USA). Recombinant GST-p53 (14-865) was purchased from Millipore (Temecula, CA, USA). Recombinant GST-IкBα (P323-30G) was purchased from SignalChem (Richmond, BC, Canada). pcDNA3.0-p53wt (69003), pCMV2-Flag- IKKβ (11103) were purchased from Addgene (Cambridge, MA, USA). pUNO1-HA-IKKβ was purchased from InvivoGen (puno1ha-hikkb). pcDNA3.0-Flag-p53 S392A was custom ordered from SignalChem. Doxorubicin (Doxo) (D2141) was purchased from Sigma (St. Louis, MO, USA). TPCA-1 (15115) was purchased from PerkinElmer (Waltham, MA, USA). Lipofectamine RNAiMAX and Lipofectamine 2000 transfection reagents were purchased from Life Technologies (Carlsbad, CA, USA). TRC human IKKβ shRNA (TRCN0000018917) was purchased from Dharmacon (Lafayetter, CO, USA) and pLKO.1 scramble (1864) was purchased from Addgene.

### Cell culture

HT1080, MIA PaCa-2, MDA-MB-231, PC3, and 293T cell lines were purchased from American Type Culture Collection (ATCC, Manassas, VA, USA). Wild-type, *Ikkα*^−/−^ or *Ikkβ*^−/−^ mouse embryonic fibroblasts (MEFs) were kindly provided by Dr. Michael Karin’s laboratory at University of California, San Diego. All cells were cultured in Dulbecco’s modified Eagle’s medium (DMEM, Corning) containing 25 mM glucose and 4mM l-glutamine supplemented with 10% fetal bovine serum (FBS), 100 U/ml penicillin, and 100 μg/ml streptomycin (Gemini Bio-Products). PC3 cells were cultured in RPMI 1640 medium supplemented with 10% FBS and 100 U/ml penicillin, and 100 μg/ml streptomycin. All cells were regularly tested for mycoplasma. For glutamine-free medium, DMEM without l-glutamine (11960, Life Technologies) was supplemented with 10% dialyzed FBS (Gemini Bio-Products). Cell viability was either determined using propidium iodide exclusion flow cytometry or using trypan blue exclusion counted by TC20 automated cell counter (Bio-Rad, Hercules, CA, USA).

### siRNA and shRNA silencing

Cells were transiently transfected according to manufacturer’s protocol with on-target SMARTpool control siRNA (D-001810-10-20) and siRNA targeting human IKKβ (L-003503-00-0005), human IKKα (L-0034733-00-0005), human p65 (L-003533-00-0005) purchased from Dharmacon. For stable gene silencing, HT1080 cells were generated via lentiviral-mediated gene transfer followed by puromycin selection.

### Quantitative real-time polymerase-chain reaction

Total RNA was extracted and purified using Trizol reagent (Life Technologies, Carlsbad, CA, USA) according to manufacturer’s guidelines. 1 μg RNA was used for qScript cDNA Synthesis Kit (Quanta Biosciences, Beverly, MA, USA). qPCR analyses were performed with SYBR Green PCR (Quanta Biosciences) using CFX Connect Real-Time PCR Detection System (Bio-Rad, Hercules, CA, USA). The following forward and reverse primers were generated to check gene expression: HUMAN (F: forward, R: reverse) ACTIN-F: 5′-CACCAACTGGGAGGACAT-3′ R: 5′-GCACAGCCT GGATAGCAAC-3′; CDKN1A-F: 5′-AGGTGGACCTGGAGACTCTCAG-3′ R: 5′-TCCTCTTGGAGAAGATCA GCCG-3′; GADD45A-F: 5′-CTGGAGGAAGTGC TCAGCAAAG-3′ R: 5′-AGAGCCACA TCTCTGTCGTCGT-3′: 18S-F 5′-CCCGTTGAACCCCATTCGTGA-3′; R: 5′-GCCTCACTAAACCATCCAATCGG-3′, MOUSE Cdkn1a-F: 5′-TCGCTGTCTTGCACTCTGGTG-3′; R: 5′-CCAATCTGCGCTTGGAGTGATAG-3′; Gadd45a-F: 5′-GCCACATCCCGGTCGTCGTC-3′; R: 5′-CGCACCATTACGGTCGGCGT-3′; 18S F: 5′-CGCTTCCTTACCTGGTTGAT-3′; R: 5′-GAGCGACCAAAGGAACCATA-3′.

### Western blotting and immunoprecipitation

Cells were lysed in buffer (50 mM Tris–HCL [pH 7.4], 5 mM sodium fluoride, 5 mM sodium pyrophosphate, 1 mM EDTA, 1 mM EGTA, 250 mM mannitol, 1% [v/v] Triton X-100) containing protease inhibitor complex (Roche) and phosphatase inhibitor (Life Technologies). Equal amounts of protein were loaded on precast NuPAGE Bis-Tris Gels (Life Technologies) followed by transfer onto nitrocellulose. For co-immunoprecipitation, one 15-cm plate containing 80% cell confluency was used for each experimental condition. Cells lysate was scrapped with lysis buffer (150 mM KCl, 0.2% [v/v] NP-40, 10% [v/v] glycerol, 20 mM Tris at pH 7.5, 0.5 mM DTT) with protease inhibitor complex (Roche) and phosphatase inhibitor (Life Technologies) on ice. Equal amounts of lysate (500 μg to 1 mg) were immunoprecipitated with 4 μg of anti-p53 (Santa Cruz). Protein G agarose or anti-Flag conjugated agarose beads (20 μl) were added to each tube and rotated at 4˚C for 24 h. Beads were washed four times with lysis buffer and resuspended in SDS loading dye and boiled for immunoblotting.

### Kinase assay

Recombinant active GST-IKKβ was incubated for 30 min at 30˚C with: kinase buffer, 200 μM ATP (Cell Signaling), and the specified recombinant proteins (GST-p53 or GST-IкBα). The reaction was quenched with 10 μl of SDS loading dye and was separated by SDS-PAGE. For phosphopeptide mass spectrometry, the reaction was repeated using the same protocol with the addition of gel drying at 80 °C for 2 h, followed by coomassie blue staining. The respective p53 band was excised from gel and sent for mass spectrometry analysis.

### Statistics

Data are represented as means ± standard deviation; statistical analysis were done in Excel. A two-tailed Student’s *t*-test was used to determine the statistical significance with *p < 0.05, **p < 0.01, ***p < 0.001.
